# Comparison of bone mineral loss by combined androgen block agonist versus GnRH in patients with prostate cancer: A 12 month-prospective observational study

**DOI:** 10.1038/srep39562

**Published:** 2017-03-06

**Authors:** Sung Han Kim, Jae Young Joung, Sohee Kim, Koon Ho Rha, Hyeong Gon Kim, Cheol Kwak, Ji Youl Lee, Seong Soo Jeon, Sung Kyu Hong, Hyeon Jeong, Moon Ki Jo, Dalsan You, In Gab Jeong, Jun Hyuk Hong, Choung-Soo Kim

**Affiliations:** 1 Department of Urology, Prostate Cancer Center, National Cancer Center, Goyang, Republic of Korea; 2 Department of Urology and Urological Science Institute, Yonsei University College of Medicine, Seoul, Republic of Korea; 3 Department of Urology, Konkuk University School of Medicine, Konkuk University Medical Center, Seoul, Republic of Korea; 4 Department of Urology, Seoul National University Hospital, Seoul, Republic of Korea; 5 Department of Urology, Seoul St. Mary’s Hospital, College of Medicine, The Catholic University of Korea, Seoul, Republic of Korea; 6 Department of Urology, Samsung Medical Center, Sungkyunkwan University School of Medicine, Seoul, Republic of Korea; 7 Department of Urology, Seoul National University Bundang Hospital, Seongnam, Republic of Korea; 8 Department of Urology, Seoul Metropolitan Government, Seoul National University Boramae Medical Center, Seoul, Republic of Korea; 9 Department of Urology, Korea Cancer Centre Hospital, Seoul, Republic of Korea; 10 Asan Medical Center, University of Ulsan College of Medicine, Seoul, Republic of Korea

## Abstract

The multi-centre, prospective, observational study was designed to examine the efficacy of continuous combined androgen block (CAB) vs. GnRH agonist monotherapy in terms of bone mineral density (BMD) change during 12 months post-androgen deprivation therapy (ADT) in Asian prostate cancer patients. Multiple regression analysis and estimated the 10-year probability of major fractures among the patients with Fracture Risk Assessment Tool were conducted to investigate the underlying factors affecting BMD. Paired t-test to evaluate the change of BMD from baseline to 12 month, and two sample t-test to examine the difference of BMD changes were used between two groups. BMD significantly decreased in both the CAB and GnRH groups, with no group wise differences. The proportion of osteopenia or osteoporosis was slightly increased after the 12-month post-ADT. Ten-year probability of hip fracture and major osteoporotic fracture was approximately 3% and 5%, respectively. In conclusion, a significant decrease of BMD by 12-month ADT was observed without any differences between the two groups, whereas ADT-related BMD loss did not induce detrimental effects on bone health in terms of increased bone fracture risk. This was the first prospective study on BMD changes as a predictor of fracture during ADT in an Asian population.

The worldwide increase in lifespan and improving medical technologies in diagnostic and therapeutic areas such as PSA screening facilitate earlier prostate cancer detection resulting in the most rapid increase in cancer among men^1^. Asian countries also showed rapid increase of prostate cancer incidence similar to Western countries, but with relatively lower incidence. The annual increase rate of prostate cancer in Korea clearly indicates that prostate cancer is the second most rapidly increasing Asian male cancer with an annual increase of 12.3% among all cancers^2^,^3^. Increasing number of early diagnosed prostate cancer cases have better prognoses, however, a significant portion of newly diagnosed patients still have advanced prostate cancer including metastatic disease and androgen deprivation therapy (ADT) is a standard treatment for such patients^4^.

The ADT comprises two different therapeutic regimens^4^. One regimen comprises either orchiectomy or gonadotropin-releasing hormone (GnRH) agonist that reduces testosterone and oestrogen concentrations; and the other of nonsteroidal androgen receptor blocker called bicalutamide that maintains testosterone and oestrogen concentrations. The bicalutamide ADT is reportedly less effective than ADT either with orchiectomy or GnRH agonist in metastatic diseases. Another combination regimen of strengthening the efficacy was the combined androgen block (CAB) with GnRH agonist plus bicalutamide, which has significant advantages over castration alone or GnRH agonist monotherapy, such as a higher proportion of patients with complete and partial responses, improved control of pain associated with metastatic disease, longer disease-free survival, and longer overall survival by an average of 3–6 months, as compared with combined treatment with the anti-androgen initiated later^4^,^5^,^6^.

However, one of the adverse effects of ADT is poor bone health due to significant decreases in bone mineral density (BMD) by 1.5–4.0% annually^7^. Combined with the underlying low BMD presented as osteopenia or osteoporosis in mostly old-aged patients with prostate cancer^8^, ADT-induced bone mineral loss that presents as osteopenia or osteoporosis is a major adverse outcome with the increasing risks of serious complications such as lumbar and hip fractures^9^,^10^,^11^. Based on the nationwide analysis of Swedish Prostate Cancer Registry, men on ADT represented an additional 30 deaths per 1000 person-years in men who develop hip fractures, as compared with an additional 20 deaths per 1000 person-years for hip fractures in the general male population^10^,^11^.

Prior experimental work showed that bone turnover markers including urinary N-telopeptide and serum osteocalcin are elevated in men receiving GnRH agonist but not in men receiving bicalutamide monotherapy, which suggests that bicalutamide monotherapy may maintain BMD and potentially prevent fracture^12^. However, comparative evaluation of BMD loss between treatment groups such as CAB and GnRH agonist monotherapy has not been previously conducted. Therefore, the study was aimed to examine the effect of CAB vs. GnRH agonist monotherapy on BMD change and to determine the underlying factors affecting BMD during 12 months post-treatment initiation in prostate cancer patients and the 10-year probability of major fractures.

## Patients and Methods

### Ethical statements

All study protocols were conducted according to the ethical guidelines of the ‘World Medical Association Declaration of Helsinki-Ethical Principles for Medical Research Involving Human Subjects’. This study was approved by the ethics committees and the Institutional Review Boards at each hospital (10 University hospitals in South Korea which were National Cancer Center, Severance Hospital, Konkuk University Medical Center, Seoul, Seoul National University Hospital, Seoul St. Mary’s Hospital, Samsung Medical Center, Seoul National University Bundang Hospital, Seoul National University Boramae Medical Center, Korea Cancer Centre Hospital, and Asan Medical center), that participated in the study, including the Institutional Review Board of the Research Institute and Hospital of Asan Medical Center (IRB No. 2010-0210), and all enrolled patients provided written informed consent.

### Study design

The study was originally designed as a non-inferiority, multi-centre (10 University hospitals in metropolitan of Seoul, South Korea which were National Cancer Center, Severance Hospital, Konkuk University Medical Center, Seoul, Seoul National University Hospital, Seoul St. Mary’s Hospital, Samsung Medical Center, Seoul National University Bundang Hospital, Seoul National University Boramae Medical Center, Korea Cancer Centre Hospital, and Asan Medical center), prospective trial on patients who were eligible for study enrolment between April 2011 and February 2014. By the statistical calculation performed by medical statistician (SK, PhD), 416 patients were needed for a one-sided type I error of 2.5% and a power of 90% with −1.5% of non-inferiority margin for change of BMD of L-spine. Assuming a 20% follow-up loss, we needed 500 patients (250 per treatment group). However, because of non-intervention and non-randomization nature, the enrolment was imbalanced between GnRH group (n = 70) and CAB group (n = 242). Data safety monitoring board (DSMB) reviewed the enrolment status and decided to stop the study earlier than planned. Hence, the limited results of this study were finally designed as a prospective, non-interventional observation cohort study. The study was also conducted in a routine clinical practice without affecting patient treatment. There is no definite standard treatment in terms of ADT options including CAB and monotherapy.

### Patients’ enrolment criteria

A total of 312 target subjects were pathologically diagnosed prostate cancer patients aged ≥50, and patients who were scheduled to receive either continuous ADT with CAB (CAB group, n = 70) or GnRH agonist monotherapy (GnRH group, N = 242) by each physician’s discretion after considering the following criteria: BMD was measured in all patients before ADT initiation. Patients who had a lowered T-score ≤−3.0 at the time of enrolment and withdrawn their consent were excluded. Patients did not receive denosumab or bisphosphonates such as pamidronate or zoledronic acid during ADT. After the 8 subjects with exclusion criteria and 51 patients with early drop-out during the study, a final of 253 patients including 196 patients in the CAB group and 57 patients in the GnRH group completed the study (Table 1).

### Bone mineral densitometry

BMD was measured by Dual energy x-ray absorptiometry (DEXA) in 10 site hospitals. The BMD of L-spine from L1-4 and total, and BMD of femur total and femur neck were measured. Using BMD values, T-score and Z-score were calculated by conventional method. The BMD values at baseline and at 12M (12^th^ month from treatment initiation) were collected at the study site.

### Fracture risk assessment

Estimation of the 10-year probability of major fractures was by the Fracture Risk Assessment Tool (FRAX^®^) with BMD. The factors included age, sex, body mass index, alcohol use, tobacco use, glucocorticoid use, rheumatoid arthritis, previous fragility fracture, family history of hip fracture, and secondary osteoporosis, and T-score of femur neck BMD.

### Statistical analysis

We evaluated the primary objective as the changes in BMD from baseline to 12-month from ADT initiation. The changes from baseline to 12-month were evaluated by paired t-test, and the difference between the treatment groups was evaluated by 2 sample t-test. Using the lowest T-score among L-spine total, femur total or femur neck at baseline and 12M, BMD status was graded as normal (T-score ≥ −1.0), osteopenia (−2.5 < T-score < −1.0), and osteoporosis (T-score ≤ −2.5)^11^. In addition, the number and proportion of subjects with osteopenia or osteoporosis were analysed with Pearson’s chi-square test. We also evaluated the underlying factors affecting BMD changes from baseline to 12M, by multiple regression analysis with treatment group, age, weight, and history of smoking and alcohol drinking as independent variables. FRAX^®^ scores of baseline, 12M and the change from baseline to 12M by treatment groups were evaluated by paired *t*-test. The difference between the treatment groups was evaluated by 2 sample *t*-test. Additionally, we evaluated the incidence of skeletal-related events (SREs) at baseline, 12M and the change from baseline to 12M by treatment group by Fisher’s exact test. We adjusted the potential effect of exercise on BMD change by using the International Physical Activity Questionnaire (IPAQ).

## Results

### Baseline characteristics

Among the 253 patients who completed the study, no differences in demographic characteristics between treatment CAB (n = 196) and GnRH (n = 57) groups were observed including occurrence of osteoporosis-related concurrent disease (p > 0.05, Table 1 and Supplementary Table 1); except for their prostate volume (52.09cc in CAB group vs. 38.84cc in GnRH group), clinical N stage (N1, 29.91% vs. 11.59%), mean daily alcohol consumption of approximately ≥2 units (40.63% vs. 22.73%) and administration of concomitant medications (41.03% vs. 52.29%) (p < 0.05, Table 1 and Supplementary Tables 1 and 2).

As for the rate of osteoporosis and osteopenia, 39.32% (92/234 subjects) of the CAB group had osteopenia and 9.40% (22/234 subjects) had osteoporosis at baseline, whereas 41.43% (29/70 subjects) of the GnRH group had osteopenia and 8.57% (6/70 subjects) had osteoporosis at baseline.

### Changes in BMD

At 12M, the proportion of the subjects with osteopenia or osteoporosis was slightly increased, but the difference between the groups was not significant (*p* = 0.3688; Table 2). As shown in Table 3, there were statistically significant mean decrease changes from baseline to 12-month in L-spine total BMD in both groups with −0.04 (SD 0.14) in the CAB group (p < 0.00090) and −0.05 (SD 0.19) in the GnRH group (p < 0.001). The rate of BMD change in L-spine also significantly decreased 2.61% in the CAB group (p < 0.009) and 4.37% in the GnRH group (p < 0.0001) with a 95% confidence interval of [−2.04%, 5.57%]. Non-inferiority of the CAB group was undetermined with the lower limit of 95% confident interval of <−1.5%, however, the group wise difference was not statistically significant (*p* = 0.1518). Furthermore, BMD significantly decreased in L1-L4 of both groups, but there were no differences between the 2 groups (p > 0.05; Supplemental Table 3).

The results showed a statistically significant decrease in L-spine total T-score in both groups with 0.34 in the CAB group and 0.43 in the GnRH group, respectively (*p* < 0.0001; Supplemental Table 4). However, the difference between the groups was not statistically significant (*p* = 0.4228).

The changes of each T-score of L1-L4 had the same pattern as total T-score. The L-spine total Z-score decreased in both groups with 0.30 in the CAB group (p < 0.0001) and 0.34 in the GnRH group (p = 0.0006), without significant differences between treatment groups (p = 0.8123; Supplemental Table 5). Likewise, the BMD of total femur and femur neck, T-score and Z-score were significantly decreased from baseline to 12M in both groups, with no significant differences between the groups (Supplemental Tables 6, 7 and 8).

### Estimated 10-year probability of major fractures

According to FRAX^®^, the mean 10-year probability of major osteoporotic fracture at 12-month was 4.96% and 5.85% in the CAB and GnRH groups, respectively, which was not significantly changed from baseline in both groups, with no significant group wise differences (p > 0.05, Table 3).

The 10-year probability of hip fracture at 12M was 1.69% and 2.41% in the CAB and GnRH groups, respectively. Significant increase from baseline by 0.20 ± 1.16% (*p* = 0.0231) was observed in the CAB group, while there was no statistically significant change over basal values in the GnRH group, and no differences between the groups (*p* = 0.5531). Two subjects in the GnRH group presented with pathologic fracture and received bone surgery with adjuvant radiation therapy at the time of participation. No new SRE occurred in both groups during ADT (Supplemental Table 9).

### Factors affecting the change in BMD

Factor analysis showed that the treatment group was not a significant risk factor for BMD change (p > 0.05, Table 4). For L-spine BMD, there were no factors affecting either T-score or Z-score among treatment groups, age, body weight, smoking and alcohol consumption (p > 0.05).

Despite the only significant effect of age on the change in L-spine BMD score (*p* = 0.0285, Table 4), the clinical effect was insignificant based on the regression estimate. For total femur, similar to L-spine, age was also a significant factor affecting the change in BMD (BMD score: *p* = 0.0445, T-score; *p* = 0.0372, Z-score: *p* = 0.0267), however, the clinical effect was insignificant (BMD score: e = 0.001, T-score: e = 0.007, Z-score: e = 0.009) (Supplemental Table 10). On the other hand, there were no factors affecting the change in BMD (BMD score, T-score, Z-score) for femur neck.

### Exercise activity

At baseline, 67.52% (158/232 subjects) in the CAB group and 91.43% (64/70 subjects) in the GnRH group responded that they performed exercise, which was significantly different (*p* < 0.0001, Supplemental Table 11). At 12-month, 69.35% (138/199 subjects) and 80.70% (46/57 subjects) in the CAB and GnRH groups, respectively maintained exercise activity, which was not significantly different (*p* = 0.0927). In order of frequency, exercise of low-intensity was most common, followed by medium- and heavy-intensity.

## Discussion

The indications for ADT in prostate cancer treatment have recently extended beyond standard use for palliation of metastatic prostate cancer^13^. Patients with ADT in adjuvant or combined therapy even in earlier stage cancers have increased, hence ADT-related side effects including bone health problems such as osteoporosis, lumbar or hip fractures by bone mineral loss are also of clinical relevance. This prospective observational study examined the effect of CAB vs. GnRH agonist monotherapy on longitudinal BMD change and calculated the 10-year probability of major fractures to determine the underlying factors affecting the ADT-related, but not tumour-related risk factors of BMD change during 12 months post-ADT initiation in prostate cancer patients.

The rapid increase of bone mineral loss and the continuous decrease of BMD within 1 year of ADT initiation are well described^14^. In the literature review of prospective studies, a wide range of BMD changes is influenced in patients who received ADT by different types of ADT, period of ADT, and patient ethnicity or presence of bone metastasis^12^,^13^,^14^,^15^,^16^,^17^,^18^,^19^,^20^,^21^. In the present study, although the degree of BMD decrease was observed in both, the CAB group and GnRH group without any statistical differences between groups (p = 0.8636, Table 2), the total BMD score of L-spine showed significantly smaller change in the CAB group, as compared to the GnRH group (p < 0.05, Table 2). Many studies on bone mineral loss in patients with prostate cancer have shown that BMD decrease of L-spine ranges from 2.40% to 4.80% post-ADT for ≤12 months, which was similar to our results of 2.58% in the CAB group and 4.37% in the GnRH group^14^,^15^,^16^,^17^,^18^,^19^,^20^,^21^,^22^,^23^.

In spite of the insignificant differences in clinical M stage and significant differences in clinical N stage (N1/M1, CAB 29.91%/23.08% vs. GnRH 11.59%13.04%, p = 0.0022/0.0711) between CAB and GnRH groups, depending on the presence of bone metastasis, a 6.6% decrease in lumbar spine BMD and a 6.5% decrease in femoral neck BMD after 6 months of CAB were observed in patients with bone metastasis^14^ (Table 1). In patients without bone metastases, 2 studies showed that BMD decreases at 12M post-ADT with GnRH agonist were 3.9% and 4.8% of total BMD of L-spine, which represents a lower degree of BMD decrease than patients with bone metastasis^19^,^22^, similar to the degree of BMD decrease in our previous results including cases without bone metastasis^8^, because the subjects with bone metastasis in our study constituted <20% of the entire study population.

Ethnic variation including genetic, hormonal or other environmental factors could potentially affect BMD change by ADT. African Americans older than 65 years have significantly higher BMD with a similar decline in BMD as compared to Caucasians^24^,^25^, which may explain the reduced incidence of hip fracture in African Americans, as compared to Caucasians^25^; whereas, another study on Jamaican men showed ADT with lower BMD^26^. Some different cross-sectional Asian studies reported that Short-term ADT of an average 23.5 months did not increase the risk of osteoporosis in Japanese prostate cancer patients and ADT was not a significant risk factor for decreased BMD^27^,^28^. Japanese men exposed to ADT have lower rates of osteoporosis with 2.3% and 8.6% in the hormone-naïve and ADT-treated patients, respectively, which was not statistically significant (p = 0.294). However, this study showed that the proportion of patients with osteopenia or osteoporosis was insignificantly increased slightly at 12M post-ADT initiation between groups (p > 0.05, Table 3) and osteoporosis was 9.4% of hormone-naïve Korean prostate cancer patients with an average age of 65.1, which is more common than the 3.4% of healthy controls in Korea (p = 0.001) and corresponding Japanese prostate cancer patients of an average age of 68.1^8^. As shown in literature reviews (Table 5), most prospective ADT studies were composed of patients from Western countries using GnRH agonists or orchiectomy, and only a few prospective studies from patients with CAB and only a few non-prospective, cross-sectional Asian studies have published their ADT-related results of bone mineral loss^27^,^28^,^29^,^30^. This study has clinical relevance because it is the first prospectively designed study of longitudinal BMD changes by different types of ADT including CAB and GnRH agonists in Asian patients.

Bicalutamide monotherapy does not facilitate any bone mineral loss, which differs from BMD change due to GnRH agonist, orchiectomy or CAB^12^. There is no prospective study, to the best of our knowledge that compares the rate of bone mineral loss depending on the types of ADT including GnRH agonist, orchiectomy or CAB. Instead, one retrospective cohort study including 742 patients presented a higher hazard ratio of fractures in patients treated with CAB than GnRH agonist, but this result may be confounded by pathologic and not ADT-related osteoporotic fractures^31^. A Jamaican cross-sectional study showed GnRH agonist, orchiectomy and CAB were a higher risk for osteoporosis than oestrogens or antiandrogen monotherapy. On adjusting for duration of therapy, the odd ratio (OR) of CAB was 9.2, as compared to oestrogens as a reference drug (p < 0.003), and the 4.5 OR of GnRH agonist (p < 0.04)^26^. This differed from our finding of no additional risk of CAB, as compared with GnRH agonist monotherapy in terms of bone mineral loss. Although the authors adjusted age and duration of ADT treatment, they did not consider various life styles such exercise activity or dietary factors that could modify BMD loss due to the limited design of cross-sectional study vs. prospective study. However, in this study, we evaluated the characteristics of patient life styles, which further strengthened the study (Tables 1 and 4, Supplementary Tables 3 and 12).

Since 2005, large database analyses found an increased risk of developing fracture within 5 years in about 20% of men starting on ADT^32^. The prevalence of osteoporosis in men on ADT has substantial variation from 9 to 53% based on the results of a meta-analysis of 13 reports published from 1999 through 2012^33^. An increased proportion of patients with osteopenia or osteoporosis were found at 12M from initiation of ADT, similar to this study, which showed slight increase at 12M post-ADT initiation between groups (p > 0.05, Table 2). However, the clinical relevance of those changes is necessarily important in terms of fracture risk related with ADT in prostate cancer. BMD measurement by DEXA is the standard test for bone health in men on ADT, but there are many concerns of its inaccurate prediction of fracture risk^34^. The FRAX score in this study developed through risk prediction algorithms that estimate fracture probability using multiple risk factors for fracture, to compensate for this limitation of BMD measurement^34^,^35^. The National Comprehensive Cancer Center Network and other published guidelines recommend screening men receiving ADT according to guidelines from the National Osteoporosis Foundation for the general population^34^. They recommend drug therapy for men with a 10 year probability of hip fracture >3% or a 10 year probability of major osteoporosis-related fracture >20%, as estimated using FRAX. In this study, the 10-year probability of hip fracture at 12M was <3%, and the 10-year probability of major osteoporotic fracture was approximately 5% (Table 3). Accordingly, the BMD loss induced by ADT for 12 months did not induce detrimental effects on bone health in terms of increased risk of bone fracture in this study.

Some limitations persist despite the prospective design including multi-centres and its non-interventional nature. Firstly, a limited number of patients completed the study and an imbalance in number of subjects per group occurred, especially the small number of subjects enrolled in GnRH group. However, the study was basically designed as a non-interventional and non-randomized study with participants arbitrarily distributed in each treatment group. Although the non-inferiority of the CAB group was not determined with statistical significance through the study, we found a significant decrease in BMD and increased proportion of osteoporosis at 12M post-ADT. Secondly, we used the FRAX score to estimate the risk of fracture. However, version of Korean FRAX^®^ has not been validated yet; hence, a Japanese database was used instead to predict the probability of bone fracture score because of the shared similarities of racial and genetic demographics between Koreans and Japanese. Thirdly, serum testosterone level was not measurable at the time of ADT start or 12M post-ADT, which was important data related to BMD of patients. Lastly, <20% of subjects with metastasis included could be confounding factors for BMD changes by ADT. Fifth, some differences in baseline characteristics such as prostate volume, clinical nodal stage, and exercise activity, which are known to be associated with the prevalence of osteoporosis, might be underlying confounding factors that were not addressed in this study. However, all information obtained from the enrolled patients might predictably indicate that the CAB and GnRH groups would not show large baseline differences in parameters related to the prevalence of osteoporosis.

However, this study was clinically meaningful due to its study design as the first prospective study of BMD change and the fracture risk during the first 12 months ADT in Asian patients, and as the first detailed information about the clinical practice of ADT in Korean Urology, which can potentially be utilized as a reference in prostate cancer patients receiving ADT. This study identified that in cases of metastatic disease, Korean urologist’s preference for ADT at a university hospital was CAB rather than GnRH agonist monotherapy for improving overall survival, as well as palliation^4^,^5^,^6^. Despite the small proportion of patients with metastatic disease who were recruited in this study, many urologists selected CAB rather than GnRH agonist monotherapy for treatment, which resulted in failure to recruit sufficient subjects in the GnRH group.

## Conclusions

A significant decrease of BMD was observed by 12 months ADT and no difference in BMD loss was found between the 2 groups. In particular, there was no clinical significance in terms of increased fracture by 12 months ADT. Based on these results, there are no safety concerns of bone-related diseases caused by additional bone loss with CAB. In addition, the data collected in this study can potentially be utilized as a reference in patients with prostate cancer receiving ADT.

## Additional Information

**How to cite this article:** Kim, S. H. *et al*. Comparison of bone mineral loss by combined androgen block agonist versus GnRH in patients with prostate cancer: A 12 month-prospective observational study. *Sci. Rep.* **7**, 39562; doi: 10.1038/srep39562 (2017).

**Publisher's note:** Springer Nature remains neutral with regard to jurisdictional claims in published maps and institutional affiliations.

## Supplementary Material

Supplementary Information

## Figures and Tables

**Table 1:** Demographic characteristics.

Parameter		CAB group N = 234		GnRH group N = 70		p-value
Age (mean ± SD, Yr)			70.70 ± 7.83		72.24 ± 8.63	0.1593^§^
(N,%)	50~59	22	9.40	5	7.14	0.1178^‡^
	60~69	69	29.49	20	28.57	
	70~79	119	50.85	30	42.86	
	≥80	24	10.26	15	21.43	
BMI (mean ± SD, kg/m^2^)		23.90	±2.91	23.77	±2.91	0.7507^§^
Past or present smoking history (N,%)	Present smoker	24	10.26	9	12.86	0.2898^†^
	Ex-smoker	88	37.61	32	45.71	
	Never smoker	122	52.14	29	41.43	
Present smoker (N,%)		24	26.69 ± 17.23	9	29.78 ± 14.59	0.6371^§^
Present non-smoker (N,%)		88	25.32 ± 21.67	32	35.41 ± 29.25	0.0714∫
Alcohol consumption (N,%)		128	54.70	44	62.86	0.2271^†^
Amount of alcohol consumption (N,%)	≥2units/day*	52	40.63	10	22.73	0.0329^†^
	<2units/day*	76	59.38	34	77.27	
Fracture history (N,%)		19	8.12	5	7.14	0.7903^†^
Family history of hip fracture (N,%)		1	0.43	0	0.00	1.0000‡
Concomitant medication (N,%)		96	41.03	38	54.29	0.0499^†^
Osteoporosis-related concurrent disease (N,%)		3	1.28	1	1.43	1.0000^‡^
initial PSA (mean ± SD, ng/mL)			213.46 ± 1287.05		30.60 ± 60.15	0.1573^∫^
Gleason score (mean ± SD)			7.76 ± 1.06		7.77 ± 1.20	0.9671^§^
Prostate volume (mean ± SD, cc)			52.09 ± 29.16		38.84 ± 16.05	0.0017^∫^
Clinical stage at diagnosis	Tx	3	1.28	0	0.00	0.0616^‡^
	T0	1	0.43	0	0.00	
	T1	30	12.82	5	7.25	
	T2	65	27.78	29	42.03	
	T3	103	44.02	32	46.38	
	T4	32	13.68	3	4.35	
	N0	164	70.09	61	88.41	0.0022^†^
	N1	70	29.91	8	11.59	
	M0	180	76.92	60	86.96	0.0711^†^
	M1	54	23.08	9	13.04	
Treatment for prostate cancer	Surgery	92	41.63	34	50.75	
	RRP	46	50.00	25	73.53	
	LRP	2	2.17	0	0.00	
	RALP	36	39.13	7	20.59	
	Other	8	8.70	2	5.88	
	Radiation	21	9.50	2	2.99	
	Primary	18	85.71	1	50.00	
	Palliative	3	14.29	1	50.00	
	None	116	52.49	32	47.76	

^§^Two sample *t*-test; ^∫^Wilcoxon’s rank sum test; ^†^Pearson’s chi-square test; ^‡^Fisher’s exact test; ^♦^Overlapped count; *Alcohol 2 units ≒ 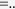 beer 1 bottle (500 mL), Korean whisky (Soju) 1/4 bottle (90 mL), Korean rice wine 1 bottle (500 mL), Wine 2 glasses (200 mL) or Whisky 2 glasses (60 mL)) RRP: radical retropubic prostatectomy; LRP: laparoscopic radical prostatectomy; RALP: robotic-assisted laparoscopic prostatectomy.

**Table 2:** Proportion of osteopenia or osteoporosis.

Time	Diagnosis	CAB group (N = 234)		GnRH group (N = 70)		*p*-value
		n	(%)	n	(%)	
Baseline	n	234		70		
	Normal (T ≥ −1.0)	120	(51.28)	35	(50.00)	0.9427†
	Osteopenia (−2.5 < T < −1.0)	92	(39.32)	29	(41.43)	
	Osteoporosis (T ≤ −2.5)	22	(9.40)	6	(8.57)	
12 month	n	186		55		
	Normal (T ≥ −1.0)	81	(43.55)	20	(36.36)	0.3688†
	Osteopenia (−2.5 < T < −1.0)	81	(43.55)	24	(43.64)	
	Osteoporosis (T ≤ −2.5)	24	(12.90)	11	(20.00)	

^†^Pearson’s chi-square test.

**Table 3:** Total bone mineral density (BMD) score at L-spine and FRAX^®^ score for major osteoporotic and hip fractures.

Variable	Time	CAB group (N = 234)		GnRH group (N = 70)		Difference 95% C.I.	Between groups *p*-value¤
**Total bone marrow density (BMD) score at L-spine**							
Changes in total BMD score		n	Mean ± SD	n	Mean ± SD		
	Baseline	223	1.16 ± 0.25	63	1.15 ± 0.22		0.8636^§^
	12 Month	184	1.13 ± 0.26	55	1.09 ± 0.19		
		176	−0.04 ± 0.14	48	−0.05 ± 0.06	0.01 (−0.03, 0.06)	0.4887^§^
	Within group *p*-value	0.0009^§§^		<0.0001^§§^			
Rate of BMD change		176	−2.61 ± 13.11	48	−4.37 ± 5.04	1.77(−2.04, 5.57)	0.1518^§^
	Within group *p*−value	0.0090^§§^		<0.0001^§§^			
**FRAX**^**®**^ **score of major osteoporotic and hip fractures**							
Major osteoporotic fracture (%)	Baseline	234	5.08 ± 3.14	70	5.35 ± 2.75		0.5194^§^
	12 month	186	4.96 ± 2.84	55	5.85 ± 3.48		
	change	186	−0.23 ± 2.79	55	0.20 ± 2.74		0.3189^§^
	Within group *p*-value	0.2635^§§^		0.5973^§§^			
Hip fracture (%)	Baseline	234	1.50 ± 1.54	70	1.87 ± 1.78		0.0943^§^
	12 month	186	1.69 ± 1.76	55	2.41 ± 2.53		
	change	186	0.20 ± 1.16	55	0.36 ± 1.97		0.5531^§^
	Within group *p*-value	0.0231^§§^		0.1798^§§§^			

Change = score at 12 month − score at baseline Rate of BMD change = (BMD score at 12 month − BMD score at baseline)/BMD score at baseline ×100 Difference = CAB group − GnRH group ^¤^ *p*-value of difference between groups (CAB group − GnRH group): ^§^Two sample *t*-test ^§§^Paired *t*-test.

**Table 4:** Factors affecting the change in L-spine BMD.

variable	factor	Estimate	Standard Error	*p*-value
BMD (g/cm^2^)	Group (1 = CAB group, 0 = GnRH group)	0.019	0.021	0.3699
	Age	0.002	0.001	**0.0285**
	Body weight	−0.001	0.001	0.3052
	Smoking history (1 = yes, 2 = no)	−0.009	0.021	0.6502
	Alcoholic history (1 = yes, 2 = no)	−0.021	0.021	0.3001
Total T-score	Group (1 = CAB group, 0 = GnRH group)	0.118	0.153	0.4432
	Age	0.014	0.008	0.0723
	Body weight	−0.004	0.007	0.5743
	Smoking history (1 = yes, 2 = no)	−0.024	0.152	0.8731
	Alcoholic history (1 = yes, 2 = no)	−0.115	0.149	0.4416
Total Z-score	Group (1 = CAB group, 0 = GnRH group)	0.046	0.156	0.7689
	Age	0.010	0.008	0.2091
	Body weight	−0.007	0.006	0.2669
	Smoking history (1 = yes, 2 = no)	−0.007	0.145	0.9617
	Alcoholic history (1 = yes, 2 = no)	−0.101	0.144	0.4851

**Table 5:** Prospective studies of bone mineral density (BMD) during androgen deprivation therapy (ADT).

Country	Stage	n	Type of ADT	Change in BMD	
Australia^14^	M1	12	CAB	−6.6% at 6 mo	L spine
Sweden^15^	M0	11	Orchiectomy	−10% at 12 mo	Proximal femur
France^16^	M0	12	GnRH agonist	−4.6% at 12 mo−3.9% at 12 mo	L spineFemoral neck
USA^17^	M0	1016	OrchiectomyGnRH agonist or CAB	−2.4% at 12 mo−3.4% at 12 mo	Femoral neck
USA^18^	M0	15	GnRH agonist	−2.8% at 12 mo	spine
Australia^19^	M0	26	GnRH agonist	−3.9% at 12 mo	L spine
USA^20^	M0	22	CAB	−3.3% at 12 mo	L spine
Italy^21^	M0	35	GnRH agonist	−2.3% at 12 mo	L spine
Spain^22^	M0	31	GnRH agonist	−4.8% at 12 mo	L spine
Australia^23^	M0	72	CAB	−1.9% at 9 mo−3.3% at 9 mo	Hipspine
Present study	M0 & M1	23470	CABGnRH agonist	−2.6% at 12 mo−4.4% at 12 mo	L spineL spine
