# Supplementary material for: Comparison of bone mineral loss by combined androgen block agonist versus GnRH in patients with prostate cancer: A 12 month-prospective observational study
Source: Sci Rep. 2017 Mar 6;7:39562. doi: 10.1038/srep39562 (PMC5338031; doi:10.1038/srep39562)
Supplement: Supplementary Information [file srep39562-s1.doc]

**Supplemental Table 1. Osteoporosis-related concurrent disease**

| **Disease** | **CAB group (N=234)** | | **GnRH group (N=70)** | | ***p*-value** |
| --- | --- | --- | --- | --- | --- |
|  | **n** | **(%)** | **n** | **(%)** |  |
| **Number^♦^** | **72** | **(30.77)** | **19** | **(27.14)** | **0.5611†** |
| Diabetes (type 1 or 2) | 40 | (17.09) | 10 | (14.29) |  |
| Mood disorder | 16 | (6.84) | 6 | (8.57) |  |
| Chemotherapy | 12 | (5.13) | 1 | (1.43) |  |
| Hyperthyroidism | 6 | (2.56) | 0 | (0.00) |  |
| Tuberculosis | 3 | (1.28) | 1 | (1.43) |  |
| Hyperparathyroidism | 3 | (1.28) | 0 | (0.00) |  |
| Atrial fibrillation | 2 | (0.85) | 2 | (2.86) |  |
| Other malignancies | 2 | (0.85) | 2 | (2.86) |  |
| Renal insufficiency | 1 | (0.43) | 1 | (1.43) |  |
| Autoimmune disease | 1 | (0.43) | 0 | (0.00) |  |
| Chronic liver diseases | 0 | (0.00) | 0 | (0.00) |  |
| Seizure disorder | 0 | (0.00) | 0 | (0.00) |  |
| Thromboembolism | 0 | (0.00) | 0 | (0.00) |  |

†: Pearson's chi-square test

♦ Overlapped count

**Supplemental Table 2** Concomitant medication

| **Anatomical main group/Therapeutic subgroup^◆^** | | **CAB group (N=234)** | | | **GnRH group (N=70)** | | |
| --- | --- | --- | --- | --- | --- | --- | --- |
|  |  | **n** | **(%)** | **[cases]** | **n** | **(%)** | **[cases]** |
| **Alimentary tract and metabolism** | | **67** | **(28.63)** | **91** | **20** | **(28.57)** | **37** |
|  | Mineral supplements | 42 | (17.95) | 42 | 10 | (14.29) | 10 |
|  | Drugs used in diabetes | 17 | (7.26) | 24 | 6 | (8.57) | 9 |
|  | Drugs for acid related disorders | 5 | (2.14) | 7 | 4 | (5.71) | 5 |
|  | Drugs for functional gastrointestinal disorders | 5 | (2.14) | 6 | 3 | (4.29) | 3 |
|  | Bile and liver therapy | 3 | (1.28) | 5 | 5 | (7.14) | 8 |
|  | Vitamins | 3 | (1.28) | 3 | 2 | (2.86) | 2 |
|  | Drugs for constipation | 2 | (0.85) | 2 | 0 | (0.00) | 0 |
|  | Antiemetics and antinauseants | 1 | (0.43) | 1 | 0 | (0.00) | 0 |
|  | Digestives, incl. Enzymes | 1 | (0.43) | 1 | 0 | (0.00) | 0 |
| **Cardiovascular system** | | **31** | **(13.25)** | **55** | **24** | **(34.29)** | **30** |
|  | Calcium channel blockers | 13 | (5.56) | 13 | 10 | (14.29) | 10 |
|  | Lipid modifying agents | 12 | (5.13) | 12 | 2 | (2.86) | 2 |
|  | Agents acting on the renin-angiotensin system | 10 | (4.27) | 10 | 3 | (4.29) | 3 |
|  | Beta blocking agents | 7 | (2.99) | 7 | 2 | (2.86) | 2 |
|  | Diuretics | 3 | (1.28) | 4 | 2 | (2.86) | 3 |
|  | Antihypertensives* | 2 | (0.85) | 2 | 0 | (0.00) | 0 |
|  | Cardiac therapy | 1 | (0.43) | 1 | 1 | (1.43) | 2 |
|  | Peripheral vasodilators | 0 | (0.00) | 0 | 1 | (1.43) | 1 |
|  | Unknown | 6 | (2.56) | 6 | 7 | (10.00) | 7 |
| **Genito urinary system and sex hormones** | | **18** | **(7.69)** | **19** | **19** | **(27.14)** | **23** |
|  | Urologicals | 18 | (7.69) | 19 | 19 | (27.14) | 23 |
| **Blood and blood forming organs** | | **15** | **(6.41)** | **16** | **9** | **(12.86)** | **9** |
|  | Antithrombotic agents | 15 | (6.41) | 16 | 9 | (12.86) | 9 |
| **Nervous system** | | **9** | **(3.85)** | **13** | **4** | **(5.71)** | **8** |
|  | Analgesics | 3 | (1.28) | 4 | 0 | (0.00) | 0 |
|  | Psychoanaleptics | 3 | (1.28) | 3 | 2 | (2.86) | 4 |
|  | Psycholeptics | 3 | (1.28) | 3 | 3 | (4.29) | 4 |
|  | Antiepileptics | 2 | (0.85) | 2 | 0 | (0.00) | 0 |
|  | Other nervous system drugs | 1 | (0.43) | 1 | 0 | (0.00) | 0 |
| **Musculo-skeletal system** | | **6** | **(2.56)** | **6** | **1** | **(1.43)** | **1** |
|  | Antiinflammatory and antirheumatic products | 3 | (1.28) | 3 | 0 | (0.00) | 0 |
|  | Antigout preparations | 2 | (0.85) | 2 | 0 | (0.00) | 0 |
|  | Muscle relaxants | 1 | (0.43) | 1 | 0 | (0.00) | 0 |
|  | Other drugs for disorders of the musculo-skeletal system | 0 | (0.00) | 0 | 1 | (1.43) | 1 |
| **Respiratory system** | | **3** | **(1.28)** | **6** | **2** | **(2.86)** | **2** |
|  | Drugs for obstructive airway diseases | 3 | (1.28) | 5 | 2 | (2.86) | 2 |
|  | Antihistamines for systemic use | 1 | (0.43) | 1 | 0 | (0.00) | 0 |
| **Systemic hormonal preparations, excl. Sex hormones and insulins** | | **2** | **(0.85)** | **2** | **1** | **(1.43)** | **1** |
|  | Corticosteroids for systemic use | 1 | (0.43) | 1 | 0 | (0.00) | 0 |
|  | Thyroid therapy | 1 | (0.43) | 1 | 0 | (0.00) | 0 |
|  | Pituitary and hypothalamic hormones and analogues | 0 | (0.00) | 0 | 1 | (1.43) | 1 |
| **Antiinfectives for systemic use** | | **0** | **(0.00)** | **0** | **1** | **(1.43)** | **5** |
|  | Antimycobacterials | 0 | (0.00) | 0 | 1 | (1.43) | 5 |
| **Antineoplastic and immunomodulating agents** | | **1** | **(0.43)** | **2** | **0** | **(0.00)** | **0** |
|  | Immunosuppressants | 1 | (0.43) | 2 | 0 | (0.00) | 0 |
| **Unknown** | | **4** | **(1.71)** | **4** | **3** | **(4.29)** | **3** |
| **Total** | | **96** | **(41.03)** | **214** | **38** | **(54.29)** | **118** |

♦ Overlapped count

* Doxazosin 2 cases

**Supplemental Table 3**. L-spine BMD: L1-L4

| **Variables** | **Time** | | **CAB group (N=234)** | | **GnRH group (N=70)** | | **Between groups *p*-value¤** |
| --- | --- | --- | --- | --- | --- | --- | --- |
| L1  (g/cm^2^) | Baseline | n | 232 | | 70 | |  |
|  |  | Mean±SD | 1.05 | ±0.21 | 1.06 | ±0.22 | 0.8156§ |
|  |  | Median | 1.03 | | 1.02 | |  |
|  |  | Min, Max | 0.50, | 1.76 | 0.66, | 1.91 |  |
|  | 12 month | n | 185 | | 55 | |  |
|  |  | Mean±SD | 1.03 | ±0.23 | 0.99 | ±0.17 |  |
|  |  | Median | 0.99 | | 0.99 | |  |
|  |  | Min, Max | 0.59, | 1.93 | 0.66, | 1.38 |  |
|  | BMD changes | n | 184 | | 55 | |  |
|  |  | Mean±SD | -0.03 | ±0.14 | -0.06 | ±0.16 | 0.2033§ |
|  |  | Median | -0.04 | | -0.05 | |  |
|  |  | Min, Max | -0.51, | 0.69 | -1.09, | 0.11 |  |
|  | *Within group p*-value | | 0.0052§§ | | 0.0103§§ | |  |
| L2  (g/cm^2^) | Baseline | n | 232 | | 70 | |  |
|  |  | Mean±SD | 1.12 | ±0.23 | 1.14 | ±0.22 | 0.5677§ |
|  |  | Median | 1.09 | | 1.13 | |  |
|  |  | Min, Max | 0.61, | 2.00 | 0.78, | 2.21 |  |
|  | 12 month | n | 185 | | 55 | |  |
|  |  | Mean±SD | 1.08 | ±0.25 | 1.07 | ±0.19 |  |
|  |  | Median | 1.04 | | 1.06 | |  |
|  |  | Min, Max | 0.61, | 1.98 | 0.70, | 1.59 |  |
|  | BMD changes | n | 184 | | 55 | |  |
|  |  | Mean±SD | -0.05 | ±0.11 | -0.05 | ±0.07 | 0.8395§ |
|  |  | Median | -0.05 | | -0.05 | |  |
|  |  | Min, Max | -0.55, | 0.50 | -0.20, | 0.18 |  |
|  | *Within group p*-value | | <0.0001§§ | | <0.0001§§ | |  |
| L3  (g/cm^2^) | Baseline | n | 231 | | 70 | |  |
|  |  | Mean±SD | 1.18 | ±0.28 | 1.19 | ±0.24 | 0.7107§ |
|  |  | Median | 1.16 | | 1.15 | |  |
|  |  | Min, Max | 0.13, | 2.53 | 0.74, | 2.13 |  |
|  | 12month | n | 185 | | 55 | |  |
|  |  | Mean±SD | 1.14 | ±0.29 | 1.14 | ±0.22 |  |
|  |  | Median | 1.11 | | 1.12 | |  |
|  |  | Min, Max | 0.57, | 2.29 | 0.68, | 1.76 |  |
|  | BMD change | n | 184 | | 55 | |  |
|  |  | Mean±SD | -0.05 | ±0.12 | -0.05 | ±0.08 | 0.9720§ |
|  |  | Median | -0.05 | | -0.05 | |  |
|  |  | Min, Max | -0.58, | 0.68 | -0.21, | 0.27 |  |
|  | *Within group p*-value | | <0.0001§§ | | <0.0001§§ | |  |
| L4  (g/cm^2^) | Baseline | n | 231 | | 70 | |  |
|  |  | Mean±SD | 1.25 | ±0.31 | 1.23 | ±0.24 | 0.5763§ |
|  |  | Median | 1.21 | | 1.22 | |  |
|  |  | Min, Max | 0.68, | 3.29 | 0.74, | 1.89 |  |
|  | 12 month | n | 184 | | 55 | |  |
|  |  | Mean±SD | 1.21 | ±0.32 | 1.16 | ±0.23 |  |
|  |  | Median | 1.16 | | 1.16 | |  |
|  |  | Min, Max | 0.66, | 2.81 | 0.70, | 1.84 |  |
|  | BMD change | n | 183 | | 55 | |  |
|  |  | Mean±SD | -0.04 | ±0.13 | -0.07 | ±0.09 | 0.0504§ |
|  |  | Median | -0.04 | | -0.06 | |  |
|  |  | Min, Max | -0.52, | 0.67 | -0.42, | 0.21 |  |
|  | *Within group p*-value | | <0.0001§§ | | <0.0001§§ | |  |

BMD change = BMD score at 12 month – BMD score at baseline

¤: *p*-value of difference between groups (CAB group – GnRH group)

§: Two sample *t*-test

§§: paired *t*-test

**Supplemental table 4**. L-spine T-score: total and L1-L4

| **Variables** | **Time** | | **CAB group (N=234)** | | **GnRH group (N=70)** | | **Between groups *p*-value¤** |
| --- | --- | --- | --- | --- | --- | --- | --- |
| Total | Baseline | n | 222 | | 63 | |  |
|  |  | Mean±SD | 0.12 | ±1.96 | -0.03 | ±1.79 | 0.5997§ |
|  |  | Median | -0.15 | | -0.40 | |  |
|  |  | Min, Max | -3.60, | 11.30 | -2.90, | 7.30 |  |
|  | 12 month | n | 184 | | 55 | |  |
|  |  | Mean±SD | -0.16 | ±2.05 | -0.57 | ±1.56 |  |
|  |  | Median | -0.50 | | -0.85 | |  |
|  |  | Min, Max | -4.50, | 8.00 | -3.50, | 4.00 |  |
|  | T-score change | n | 175 | | 48 | |  |
|  |  | Mean±SD | -0.34 | ±1.00 | -0.43 | ±0.53 | 0.4228§ |
|  |  | Median | -0.40 | | -0.40 | |  |
|  |  | Min, Max | -4.30, | 5.57 | -2.10, | 1.40 |  |
|  | Within group *p*-value | | <0.0001§§ | | <0.0001§§ | |  |
| L1 | Baseline | n | 232 | | 70 | |  |
|  |  | Mean±SD | -0.20 | ±1.72 | -0.36 | ±1.68 | 0.4964§ |
|  |  | Median | -0.40 | | -0.55 | |  |
|  |  | Min, Max | -3.70, | 5.30 | -3.20, | 6.70 |  |
|  | 12month | n | 185 | | 55 | |  |
|  |  | Mean±SD | -0.42 | ±1.87 | -0.76 | ±1.51 |  |
|  |  | Median | -0.80 | | -0.80 | |  |
|  |  | Min, Max | -4.30, | 6.90 | -3.60, | 2.60 |  |
|  | T-score change | n | 184 | | 55 | |  |
|  |  | Mean±SD | -0.29 | ±1.03 | -0.26 | ±0.68 | 0.7946§ |
|  |  | Median | -0.40 | | -0.30 | |  |
|  |  | Min, Max | -4.20, | 5.20 | -1.80, | 2.50 |  |
|  | Within group *p*-value | | 0.0002§§ | | 0.0063§§ | |  |
| L2 | Baseline | n | 232 | | 70 | |  |
|  |  | Mean±SD | -0.20 | ±1.81 | -0.24 | ±1.84 | 0.8888§ |
|  |  | Median | -0.40 | | -0.50 | |  |
|  |  | Min, Max | -3.60, | 6.70 | -3.50, | 8.50 |  |
|  | 12 month | n | 185 | | 55 | |  |
|  |  | Mean±SD | -0.53 | ±1.98 | -0.79 | ±1.59 |  |
|  |  | Median | -0.80 | | -1.00 | |  |
|  |  | Min, Max | -7.00, | 6.60 | -3.60, | 3.30 |  |
|  | T-score change | n | 184 | | 55 | |  |
|  |  | Mean±SD | -0.41 | ±1.06 | -0.42 | ±0.63 | 0.9559§ |
|  |  | Median | -0.40 | | -0.50 | |  |
|  |  | Min, Max | -6.70, | 4.20 | -2.10, | 1.50 |  |
|  | Within group *p*-value | | <0.0001§§ | | <0.0001§§ | |  |
| L3 | Baseline | n | 231 | | 70 | |  |
|  |  | Mean±SD | 0.21 | ±2.10 | 0.15 | ±1.97 | 0.8288§ |
|  |  | Median | -0.20 | | -0.40 | |  |
|  |  | Min, Max | -3.90, | 12.30 | -2.70, | 7.80 |  |
|  | 12month | n | 185 | | 55 | |  |
|  |  | Mean±SD | -0.10 | ±2.24 | -0.32 | ±1.74 |  |
|  |  | Median | -0.50 | | -0.50 | |  |
|  |  | Min, Max | -4.60, | 10.30 | -3.40, | 4.70 |  |
|  | T-score change | n | 184 | | 55 | |  |
|  |  | Mean±SD | -0.39 | ±0.96 | -0.39 | ±0.67 | 0.9852§ |
|  |  | Median | -0.40 | | -0.50 | |  |
|  |  | Min, Max | -4.90, | 5.70 | -2.10, | 2.20 |  |
|  | Within group *p*-value | | <0.0001§§ | | <0.0001§§ | |  |
| L4 | Baseline | n | 231 | | 70 | |  |
|  |  | Mean±SD | 0.75 | ±2.45 | 0.40 | ±1.85 | 0.2045§ |
|  |  | Median | 0.30 | | 0.15 | |  |
|  |  | Min, Max | -3.50, | 18.70 | -2.90, | 5.80 |  |
|  | 12month | n | 184 | | 55 | |  |
|  |  | Mean±SD | 0.40 | ±2.55 | -0.17 | ±1.83 |  |
|  |  | Median | 0.00 | | -0.40 | |  |
|  |  | Min, Max | -4.40, | 14.80 | -3.40, | 5.40 |  |
|  | T-score change | n | 183 | | 55 | |  |
|  |  | Mean±SD | -0.35 | ±1.06 | -0.54 | ±0.79 | 0.1682§ |
|  |  | Median | -0.30 | | -0.50 | |  |
|  |  | Min, Max | -4.40, | 5.50 | -3.50, | 1.70 |  |
|  | within *p*-value | | <0.0001§§ | | <0.0001§§ | |  |

Change = score at 12 month – score at baseline

¤: *p*-value of difference between groups (CAB group – GnRH group)

§: Two sample *t*-test

§§: paired *t*-test

**Supplemental table 5**. L-spine Z-score: Total and L1-L4

| **Variables** | **Time** | | **CAB group (N=234)** | | **GnRH group (N=70)** | | **Between groups *p*-value¤** |
| --- | --- | --- | --- | --- | --- | --- | --- |
| Total | Baseline | n | 215 | | 52 | |  |
|  |  | Mean±SD | 1.03 | ±1.74 | 0.90 | ±1.69 | 0.6246§ |
|  |  | Median | 0.70 | | 0.85 | |  |
|  |  | Min, Max | -2.50, | 7.70 | -2.20, | 8.10 |  |
|  | 12 month | n | 184 | | 54 | |  |
|  |  | Mean±SD | 0.79 | ±1.92 | 0.54 | ±1.52 |  |
|  |  | Median | 0.35 | | 0.50 | |  |
|  |  | Min, Max | -2.60, | 8.70 | -2.80, | 5.80 |  |
|  | change | n | 168 | | 37 | |  |
|  |  | Mean±SD | -0.30 | ±0.90 | -0.34 | ±0.54 | 0.8123§ |
|  |  | Median | -0.30 | | -0.40 | |  |
|  |  | Min, Max | -3.80, | 4.80 | -1.20, | 1.20 |  |
|  | Within group *p*-value | | <0.0001§§ | | 0.0006§§ | |  |
| L1 | Baseline | n | 232 | | 69 | |  |
|  |  | Mean±SD | 0.74 | ±1.54 | 0.71 | ±1.58 | 0.8903§ |
|  |  | Median | 0.45 | | 0.70 | |  |
|  |  | Min, Max | -2.50, | 6.10 | -2.90, | 7.50 |  |
|  | 12 month | n | 185 | | 54 | |  |
|  |  | Mean±SD | 0.52 | ±1.79 | 0.29 | ±1.42 |  |
|  |  | Median | 0.10 | | 0.45 | |  |
|  |  | Min, Max | -2.50, | 7.80 | -3.40, | 4.40 |  |
|  | Change | n | 184 | | 54 | |  |
|  |  | Mean±SD | -0.26 | ±0.91 | -0.32 | ±0.62 | 0.6514§ |
|  |  | Median | -0.30 | | -0.30 | |  |
|  |  | Min, Max | -3.00, | 4.40 | -2.00, | 1.00 |  |
|  | Within group *p*-value | | 0.0002§§ | | 0.0004§§ | |  |
| L2 | Baseline | n | 232 | | 69 | |  |
|  |  | Mean±SD | 0.76 | ±1.65 | 0.85 | ±1.78 | 0.7192§ |
|  |  | Median | 0.50 | | 0.60 | |  |
|  |  | Min, Max | -2.60, | 7.50 | -2.20, | 9.30 |  |
|  | 12 month | n | 185 | | 54 | |  |
|  |  | Mean±SD | 0.47 | ±1.84 | 0.31 | ±1.53 |  |
|  |  | Median | 0.00 | | 0.45 | |  |
|  |  | Min, Max | -3.00, | 7.40 | -3.30, | 5.10 |  |
|  | Change | n | 184 | | 54 | |  |
|  |  | Mean±SD | -0.34 | ±0.92 | -0.44 | ±0.69 | 0.4722§ |
|  |  | Median | -0.30 | | -0.45 | |  |
|  |  | Min, Max | -5.20, | 3.40 | -2.30, | 1.30 |  |
|  | 군내 *p*-value | | <0.0001§§ | | <0.0001§§ | |  |
| L3 | Baseline | n | 231 | | 69 | |  |
|  |  | Mean±SD | 1.19 | ±1.85 | 1.23 | ±1.95 | 0.8987§ |
|  |  | Median | 0.90 | | 1.00 | |  |
|  |  | Min, Max | -2.40, | 8.50 | -2.10, | 8.70 |  |
|  | 12 month | n | 185 | | 54 | |  |
|  |  | Mean±SD | 0.88 | ±2.07 | 0.81 | ±1.73 |  |
|  |  | Median | 0.40 | | 0.75 | |  |
|  |  | Min, Max | -2.70, | 9.90 | -2.50, | 6.50 |  |
|  | Change | n | 184 | | 54 | |  |
|  |  | Mean±SD | -0.35 | ±0.91 | -0.38 | ±0.73 | 0.8543§ |
|  |  | Median | -0.40 | | -0.50 | |  |
|  |  | Min, Max | -3.70, | 4.80 | -2.20, | 2.00 |  |
|  | Within group *p*-value | | <0.0001§§ | | 0.0004§§ | |  |
| L4 | Baseline | n | 231 | | 69 | |  |
|  |  | Mean±SD | 1.67 | ±2.14 | 1.52 | ±1.87 | 0.6053§ |
|  |  | Median | 1.30 | | 1.30 | |  |
|  |  | Min, Max | -2.00, | 12.40 | -2.00, | 7.70 |  |
|  | 12 month | n | 184 | | 54 | |  |
|  |  | Mean±SD | 1.33 | ±2.32 | 0.98 | ±1.77 |  |
|  |  | Median | 0.95 | | 0.80 | |  |
|  |  | Min, Max | -2.70, | 11.20 | -1.90, | 7.20 |  |
|  | Change | n | 183 | | 54 | |  |
|  |  | Mean±SD | -0.30 | ±0.97 | -0.54 | ±0.85 | 0.0968§ |
|  |  | Median | -0.30 | | -0.50 | |  |
|  |  | Min, Max | -3.50, | 4.70 | -3.70, | 1.50 |  |
|  | Within group *p*-value | | <0.0001§§ | | <0.0001§§ | |  |

Change = score at 12 month – score at baseline

¤: *p*-value of difference between groups (CAB group – GnRH group)

§: Two sample *t*-test

§§: paired *t*-test

**Supplemental Table 6. BMD of total femur and femur neck**

| **Variables** | **Time** | | **CAB group (N=234)** | | **GnRH group (N=70)** | | **Difference  95% C.I.** | **Between groups *p*-value¤** |
| --- | --- | --- | --- | --- | --- | --- | --- | --- |
| Total  (g/cm^2^) | Baseline | n | 234 | | 70 | |  |  |
|  |  | Mean±SD | 0.96 | ±0.15 | 0.97 | ±0.17 |  | 0.4979§ |
|  |  | Median | 0.94 | | 0.99 | |  |  |
|  |  | Min, Max | 0.64, | 1.58 | 0.64, | 1.90 |  |  |
|  | 12 month | n | 186 | | 55 | |  |  |
|  |  | Mean±SD | 0.92 | ±0.15 | 0.92 | ±0.13 |  |  |
|  |  | Median | 0.92 | | 0.94 | |  |  |
|  |  | Min, Max | 0.52, | 1.39 | 0.63, | 1.24 |  |  |
|  | Change | n | 186 | | 55 | |  |  |
|  |  | Mean±SD | -0.04 | ±0.06 | -0.04 | ±0.04 | 0.00 [-0.02, 0.02] | 0.9437§ |
|  |  | Median | -0.03 | | -0.04 | |  |  |
|  |  | Min, Max | -0.29, | 0.30 | -0.23, | 0.02 |  |  |
|  | Within group *p*-value | | <0.0001§§ | | <0.0001§§ | |  |  |
| Neck  (g/cm^2^) | Baseline | n | 234 | | 70 | |  |  |
|  |  | Mean±SD | 0.85 | ±0.16 | 0.85 | ±0.12 |  | 0.8907§ |
|  |  | Median | 0.84 | | 0.85 | |  |  |
|  |  | Min, Max | 0.53, | 1.54 | 0.62, | 1.14 |  |  |
|  | 12 month | n | 186 | | 55 | |  |  |
|  |  | Mean±SD | 0.83 | ±0.16 | 0.81 | ±0.14 |  |  |
|  |  | Median | 0.83 | | 0.83 | |  |  |
|  |  | Min, Max | 0.44, | 1.27 | 0.47, | 1.16 |  |  |
|  | Change | n | 186 | | 55 | |  |  |
|  |  | Mean±SD | -0.02 | ±0.06 | -0.04 | ±0.06 | 0.01 [0.00, 0.03] | 0.1442§ |
|  |  | Median | -0.02 | | -0.03 | |  |  |
|  |  | Min, Max | -0.25, | 0.43 | -0.23, | 0.12 |  |  |
|  | Within group *p*-value | | <0.0001§§ | | <0.0001§§ | |  |  |

Change = score at 12 month – score at baseline

¤: *p*-value of difference between groups (CAB group – GnRH group)

§: Two sample *t*-test

§§: paired *t*-test

**Supplemental table 7. T-score of total femur and femur neck**

| **Variable** | **Time** | | **CAB group (N=234)** | | **GnRH group (N=70)** | | **Difference  95% C.I.** | **Between groups *p*-value¤** |
| --- | --- | --- | --- | --- | --- | --- | --- | --- |
| Total | Baseline | n | 234 | | 70 | |  |  |
|  |  | Mean±SD | 0.05 | ±1.19 | 0.05 | ±1.11 |  | 0.9880§ |
|  |  | Median | 0.00 | | 0.25 | |  |  |
|  |  | Min, Max | -2.70, | 4.90 | -2.50, | 2.30 |  |  |
|  | 12 month | n | 186 | | 55 | |  |  |
|  |  | Mean±SD | -0.18 | ±1.16 | -0.25 | ±1.08 |  |  |
|  |  | Median | -0.11 | | 0.00 | |  |  |
|  |  | Min, Max | -2.90, | 3.50 | -2.90, | 2.30 |  |  |
|  | Change | n | 186 | | 55 | |  |  |
|  |  | Mean±SD | -0.27 | ±0.45 | -0.26 | ±0.33 | -0.01 [-0.14, 0.12] | 0.8835§ |
|  |  | Median | -0.30 | | -0.30 | |  |  |
|  |  | Min, Max | -2.10, | 2.20 | -1.70, | 0.70 |  |  |
|  | Within group *p*-value | | <0.0001§§ | | <0.0001§§ | |  |  |
| Neck | Baseline | n | 234 | | 70 | |  |  |
|  |  | Mean±SD | -0.63 | ±1.18 | -0.69 | ±0.97 |  | 0.7177§ |
|  |  | Median | -0.70 | | -0.65 | |  |  |
|  |  | Min, Max | -3.00, | 4.60 | -2.90, | 1.50 |  |  |
|  | 12 month | n | 186 | | 55 | |  |  |
|  |  | Mean±SD | -0.75 | ±1.14 | -0.97 | ±1.09 |  |  |
|  |  | Median | -0.80 | | -0.80 | |  |  |
|  |  | Min, Max | -3.20, | 2.40 | -3.80, | 1.60 |  |  |
|  | Change | n | 186 | | 55 | |  |  |
|  |  | Mean±SD | -0.16 | ±0.48 | -0.21 | ±0.34 | 0.04 [-0.09, 0.18] | 0.5229§ |
|  |  | Median | -0.20 | | -0.20 | |  |  |
|  |  | Min, Max | -2.00, | 3.50 | -1.00, | 0.90 |  |  |
|  | Within group *p*-value | | <0.0001§§ | | <0.0001§§ | |  |  |

Change = score at 12 month – score at baseline

¤: *p*-value of difference between groups (CAB group – GnRH group)

§: Two sample *t*-test

§§: paired *t*-test

**Supplemental table 8. Z-score of total femur and femur neck**

| **Variable** | **Time** | | | **CAB group (N=234)** | | **GnRH group (N=70)** | | **Difference  95% C.I.** | **Between groups *p*-value¤** |
| --- | --- | --- | --- | --- | --- | --- | --- | --- | --- |
| Total | Baseline | n | | 233 | | 69 | |  |  |
|  |  | Mean±SD | | 1.05 | ±1.20 | 1.10 | ±1.03 |  | 0.8016§ |
|  |  | Median | | 0.90 | | 1.10 | |  |  |
|  |  | Min, Max | | -1.30, | 5.80 | -1.30, | 3.30 |  |  |
|  | 12 month | n | | 186 | | 54 | |  |  |
|  |  | Mean±SD | | 0.79 | ±1.14 | 0.88 | ±0.95 |  |  |
|  |  | Median | | 0.80 | | 1.00 | |  |  |
|  |  | Min, Max | | -1.70, | 4.50 | -1.20, | 3.10 |  |  |
|  | Change | n | | 185 | | 54 | |  |  |
|  |  | Mean±SD | | -0.28 | ±0.53 | -0.21 | ±0.39 | -0.07 [-0.22, 0.08] | 0.3778§ |
|  |  | Median | | -0.30 | | -0.20 | |  |  |
|  |  | Min, Max | | -4.00, | 2.20 | -1.20, | 1.40 |  |  |
|  | Within group *p*-value | | | <0.0001§§ | | 0.0002§§ | |  |  |
| Neck | Baseline | | n | 233 | | 69 | |  |  |
|  |  |  | Mean±SD | 0.76 | ±1.10 | 0.75 | ±0.88 |  | 0.9375§ |
|  |  |  | Median | 0.60 | | 0.60 | |  |  |
|  |  |  | Min, Max | -1.20, | 5.70 | -1.40, | 2.40 |  |  |
|  | 12 month | | n | 186 | | 54 | |  |  |
|  |  |  | Mean±SD | 0.62 | ±1.07 | 0.54 | ±0.98 |  |  |
|  |  |  | Median | 0.55 | | 0.60 | |  |  |
|  |  |  | Min, Max | -1.70, | 3.80 | -2.00, | 2.90 |  |  |
|  | Change | | n | 185 | | 54 | |  |  |
|  |  |  | Mean±SD | -0.16 | ±0.50 | -0.18 | ±0.36 | 0.02 [-0.12, 0.17] | 0.7167§ |
|  |  |  | Median | -0.20 | | -0.20 | |  |  |
|  |  |  | Min, Max | -1.80, | 3.50 | -0.90, | 0.90 |  |  |
|  | Within group *p*-value | | | <0.0001§§ | | 0.0006§§ | |  |  |

Change= score at 12 month – score at baseline

¤: *p*-value of difference between groups (CAB group – GnRH group)

§: Two sample *t*-test

§§: paired *t*-test

Supplemental Table 9. Skeletal-related events

| **Time** | **Variable** | **CAB group (N=234)** | | **GnRH group (N=70)** | | ***p*-value** |
| --- | --- | --- | --- | --- | --- | --- |
|  |  | **n** | **(%)** | **n** | **(%)** |  |
| Baseline | n | 234 | | 70 | |  |
|  | None | 234 | (100.00) | 69 | (98.57) | 0.2303‡ |
|  | present | 0 | (0.00) | 1 | (1.43) |  |
|  | Pathologic fracture | 0 | (0.00) | 1 | (1.43) |  |
|  | Spinal cord compression | 0 | (0.00) | 0 | (0.00) |  |
|  | Palliative radiation or surgery for bone metastasis | 0 | (0.00) | 1 | (1.43) |  |
| 12 month | N | 199 | | 57 | |  |
|  | None | 199 | (100.00) | 57 | (100.00) | - |
|  | present | 0 | (0.00) | 0 | (0.00) |  |
|  | Pathologic fracture | 0 | (0.00) | 0 | (0.00) |  |
|  | Spinal cord compression | 0 | (0.00) | 0 | (0.00) |  |
|  | Palliative radiation or surgery for bone metastasis | 0 | (0.00) | 0 | (0.00) |  |

‡: Fisher's exact test

**Supplemental table 10. Factors affecting the change in femur BMD**

| **variable** | | **factor** | **Estimate** | **Standard Error** | ***p*-value** |
| --- | --- | --- | --- | --- | --- |
| BMD (g/cm^2^) | Total | Group (1=CAB group, 0=GnRH group) | 0.002 | 0.008 | 0.8448 |
|  |  | Age | 0.001 | 0.000 | **0.0445** |
|  |  | Body weight | 0.000 | 0.000 | 0.7424 |
|  |  | Smoking history (1 = yes, 2 = no) | -0.013 | 0.008 | 0.1185 |
|  |  | Alcoholic history (1 = yes, 2 = no) | 0.005 | 0.008 | 0.5318 |
|  | Neck | Group (1=CAB group, 0=GnRH group) | 0.013 | 0.010 | 0.1799 |
|  |  | Age | 0.000 | 0.001 | 0.7817 |
|  |  | Body weight | 0.000 | 0.000 | 0.9773 |
|  |  | Smoking history (1 = yes, 2 = no) | -0.006 | 0.010 | 0.5379 |
|  |  | Alcoholic history (1 = yes, 2 = no) | 0.007 | 0.010 | 0.4752 |
| T-score | Total | Group (1=CAB group, 0=GnRH group) | 0.010 | 0.066 | 0.8797 |
|  |  | Age | 0.007 | 0.004 | **0.0372** |
|  |  | Body weight | -0.002 | 0.003 | 0.5808 |
|  |  | Smoking history (1 = yes, 2 = no) | -0.047 | 0.066 | 0.4807 |
|  |  | Alcoholic history (1 = yes, 2 = no) | 0.037 | 0.065 | 0.5728 |
|  | Neck | Group (1=CAB group, 0=GnRH group) | 0.034 | 0.071 | 0.6317 |
|  |  | Age | 0.000 | 0.004 | 0.9572 |
|  |  | Body weight | 0.000 | 0.003 | 0.9639 |
|  |  | Smoking history (1 = yes, 2 = no) | -0.103 | 0.071 | 0.1478 |
|  |  | Alcoholic history (1 = yes, 2 = no) | 0.082 | 0.070 | 0.2403 |
| Z-score | Total | Group (1=CAB group, 0=GnRH group) | -0.055 | 0.078 | 0.4841 |
|  |  | Age | 0.009 | 0.004 | **0.0267** |
|  |  | Body weight | 0.001 | 0.004 | 0.7466 |
|  |  | Smoking history (1 = yes, 2 = no) | -0.075 | 0.078 | 0.3383 |
|  |  | Alcoholic history (1 = yes, 2 = no) | 0.030 | 0.077 | 0.7013 |
|  | Neck | Group (1=CAB group, 0=GnRH group) | 0.014 | 0.075 | 0.8486 |
|  |  | Age | -0.002 | 0.004 | 0.5780 |
|  |  | Body weight | -0.001 | 0.004 | 0.8432 |
|  |  | Smoking history (1 = yes, 2 = no) | -0.075 | 0.075 | 0.3176 |
|  |  | Alcoholic history (1 = yes, 2 = no) | 0.108 | 0.074 | 0.1453 |

**Supplemental table 11.** Exercise activity

| **Time** | **Intensity** | **CAB group (N=234)** | | **GnRH group (N=70)** | | ***p*-value** |
| --- | --- | --- | --- | --- | --- | --- |
|  |  | **n** | **(%)** | **n** | **(%)** |  |
| Baseline | N | 234 | | 70 | |  |
|  | No | 76 | (32.48) | 6 | (8.57) | <0.0001† |
|  | Yes | 158 | (67.52) | 64 | (91.43) |  |
|  | Low | 95 | (60.13) | 47 | (73.44) |  |
|  | Medium | 46 | (29.11) | 12 | (18.75) |  |
|  | heavy | 17 | (10.76) | 5 | (7.81) |  |
| 12 month | N | 199 | | 57 | |  |
|  | No | 61 | (30.65) | 11 | (19.30) | 0.0927† |
|  | Yes | 138 | (69.35) | 46 | (80.70) |  |
|  | Low | 95 | (68.84) | 31 | (67.39) |  |
|  | Medium | 31 | (22.46) | 10 | (21.74) |  |
|  | heavy | 12 | (8.70) | 5 | (10.87) |  |

†: Pearson's chi-square test
